# A rapid review of nutrition and exercise approaches to managing unintentional weight loss, muscle loss, and malnutrition in cancer

**DOI:** 10.1093/oncolo/oyae261

**Published:** 2024-10-08

**Authors:** Brenton J Baguley, Lara Edbrooke, Linda Denehy, Carla M Prado, Nicole Kiss

**Affiliations:** Institute for Physical Activity and Nutrition, Deakin University, Geelong, VIC 3125, Australia; Allied Health Research, Peter MacCallum Cancer Centre, Melbourne, VIC 3051, Australia; Health Services Research Department, Peter MacCallum Cancer Centre, Melbourne, VIC 3051, Australia; Physiotherapy Department, The University of Melbourne, Melbourne, VIC 3052, Australia; Health Services Research Department, Peter MacCallum Cancer Centre, Melbourne, VIC 3051, Australia; Physiotherapy Department, The University of Melbourne, Melbourne, VIC 3052, Australia; Department of Agricultural, Food and Nutritional Science, University of Alberta, Edmonton, AB T6G 2R3, Canada; Institute for Physical Activity and Nutrition, Deakin University, Geelong, VIC 3125, Australia; Allied Health Research, Peter MacCallum Cancer Centre, Melbourne, VIC 3051, Australia

**Keywords:** nutrition, exercise, multimodal interventions, unintentional weight loss, malnutrition

## Abstract

**Purpose:**

This narrative review summarizes the evidence for nutrition, exercise, and multimodal interventions to maintain weight and muscle mass and prevent malnutrition from meta-analysis, systematic reviews, and randomized controlled trials published within the last 5 years, and in comparison to future research priority areas identified by international guidelines.

**Recent findings:**

Dietary counseling with oral nutrition support (ONS), escalated to enteral nutrition if weight loss continues, is the gold standard treatment approach to maintaining weight and preventing malnutrition. Recent ONS trials with dietary counseling show promising findings for weight maintenance, extending the literature to include studies in chemoradiotherapy, however, change in body composition is rarely evaluated. Emerging trials have evaluated the impact of isolated nutrients, amino acids, and their derivatives (ie, β-hydroxy β-methylbutyrate) on muscle mass albeit with mixed effects. There is insufficient evidence evaluating the effect of exercise interventions on unintentional weight loss, muscle mass, and malnutrition, however, our knowledge of the impact of multimodal nutrition and exercise interventions is advancing. Prehabilitation interventions may attenuate weight and muscle loss after surgery, particularly for patients having gastrointestinal and colorectal surgery. Multimodal trials that commence during treatment show mixed effects on weight and muscle mass when measured.

**Summary:**

This review highlights that the evidence for preventing unintentional weight loss and malnutrition from cancer treatment is strong within nutrition. Multimodal interventions are emerging as effective interventions to prevent unintentional weight loss. Promising interventions are demonstrating improvements in muscle mass, however further exploration through studies designed to determine the effect on muscle is required.

## Teaser

This narrative review summarizes the evidence for nutrition, exercise, and multimodal interventions to maintain weight and muscle mass and prevent malnutrition within the last 5 years.

**Implications for practice summary**

Early and regular dietary counseling with or without oral nutrition support (ONS) remains the front-line approach to managing, treating, and preventing unintentional weight and muscle loss and malnutrition. There is emerging evidence of the cost-effectiveness of structured nutrition protocols to prove individualized nutrition support to hospitalized patients with cancer at risk of malnutrition. Exercise interventions are safe and can prevent muscle loss across the cancer treatment continuum. Given the breadth of evidence across multiple cancers and treatments multimodal rehabilitation should be included for hospitalized cancer patients at risk or with malnutrition. Prehabilitation trials are emerging but offer safe and effective interventions to offset weight and muscle loss post-surgery.

## Introduction

Cancer-related malnutrition is common in people prior to, undergoing and recovering from cancer treatment, affecting approximately one in 3 people, albeit with a higher prevalence in people with cancers of the head and neck, lung, and gastrointestinal tract.^1,2^ Global diagnostic criteria for malnutrition consider 3 phenotypic criteria, unintentional loss of weight, reduced muscle mass, and low body mass index (BMI) alongside 2 etiologic criteria, reduced food intake, and systemic inflammation.^3,4^ At least 1 phenotypic and 1 etiologic criterion must be met for a diagnosis of malnutrition.^3,4^ Malnutrition and its components, unintentional weight loss, and reduced muscle mass, are each independently associated with serious adverse outcomes in people with cancer including early mortality and reduced quality of life.^5-7^ Importantly, reduced muscle mass can occur independently of malnutrition, with studies demonstrating only a 20% overlap between these conditions in people with cancer.^8,9^ Furthermore, weight stability may mask underlying muscle loss, with an observational study in people with colorectal cancer found that 1 in 8 participants developed low muscle mass despite remaining weight stable.^10^ Therefore, a comprehensive nutritional assessment, that considers both recent unintentional weight loss and muscle mass, as well as food intake, symptoms, social factors, and functional status, is important to guide the initiation of appropriate intervention by a multidisciplinary team of health professionals. Validated tools such as the Patient-Generated Subjective Global Assessment (PG-SGA) may be used and supported by further measures of muscle mass suitable for use in clinical practice, such as calf or mid-upper arm circumference or bioelectrical impendence analysis.^4^

Nutrition and exercise are effective evidence-based treatments for cancer-related malnutrition and muscle loss, as recommended in guidelines from the European Society for Clinical Nutrition and Metabolism (ESPEN),^11,12^ Clinical Oncology Society of Australia (COSA),^13^ and the American Society for Clinical Oncology (ASCO).^14^ Current recommendations for nutrition center around providing individualized dietary advice and oral nutrition supplements (ONS) to support adequate nutritional intake and improve quality of life, escalating to enteral (EN), or parental nutrition (PN) as required.^11,13^ Recommendations for the use of special purpose nutrients vary, with omega-3 fatty acids recommended to improve appetite, muscle mass, and body weight, however, the evidence to support the use of amino acids and metabolites such as β-hydroxy β-methylbutyrate (HMB) is currently deemed insufficient.^11^ Exercise is recognized within the ESPEN and COSA guidelines as necessary to support muscle mass, strength, and function, with resistance exercise required, in addition to aerobic exercise, to maintain muscle mass and strength.^11,13^ However, the ASCO guidelines recommend aerobic and resistance exercise to mitigate the impact of treatment side effects albeit noting that studies testing the effect of exercise on body composition have been predominantly reported in people with breast or prostate cancer with variable effects.^14^

Several key areas of focus for future research are identified in the current guidelines. While evidence for the positive effect of individualized dietary counseling with or without ONS on improving dietary intake and quality of life is established, the ESPEN guidelines suggest further investigation of the effect on clinical outcomes such as treatment tolerance and survival is required.^11^ Similarly, further research is to determine whether enteral nutrition, parenteral nutrition, or combinations affect clinical outcomes in those patients unable to achieve adequate food intake through dietary counseling and ONS.^11^ With regard to special purpose nutrients, there is a call for large randomized trials (RCTs) to investigate HMB in weight loss patients and to examine whether omega-3 fatty acids may impact clinical outcomes such as survival when provided alongside cancer treatment.^11^ The need to establish an effect on clinical outcomes is echoed in areas for future research in exercise, with a need to investigate the effect of exercise in people with malnutrition and muscle loss on clinical outcomes at all stages of treatment as well as examine the differential and combined effect of aerobic and resistance exercise.^11^ Furthermore, there is a potential role for both nutrition and exercise interventions focused on maintaining muscle mass to support the maintenance of optimal chemotherapy treatment since the impact of low muscle mass on dose-limiting treatment toxicities is well established.^14^ The ASCO guidelines highlight a particular need for studies to examine the potential health economic benefit of nutrition and exercise interventions throughout cancer treatment from potential reductions in healthcare utilization.^14^ In this brief narrative review, we will summarize the progress made over the past 5 years in these areas with a focus on RCTs, systematic reviews, and meta-analyses. Interventions included nutrition, exercise, and multimodal interventions that measured change in body weight, body composition (muscle and fat mass), and malnutrition across any stage of the cancer continuum (pre-, during-, and post-treatment). Studies specifically in cachexia were included if participant criteria for inclusion in the study defined cachexia as unintentional weight loss.

## Nutrition approaches to unintentional weight loss, muscle, and malnutrition

### Dietary counseling and structured escalation of nutrition support

Interventions published in the past 5 years that examined the effect of providing individualized dietary counseling, including ONS, enteral, or parenteral support are summarized in Table 1. Nutrition interventions show mixed findings for maintaining or increasing weight during cancer treatment through dietary interventions specifically in head and neck,^16^ gastrointestinal,^18,19,23^ colorectal,^15,22^ and mixed cancers^17,20,21^ where malnutrition risk is high. In addition, a limited number of trials have included measures of body composition, hindering our understanding of the best nutrition strategy to address muscle loss, and other clinical outcomes, such as severe treatment toxicity and completion. Overall, the evidence from systematic reviews is also mixed. A systematic review and meta-analysis of 6 RCTs reported improvement in energy intake but not body weight (WMD = 4.28 kg; *P* = .06) from dietary counseling in a mixed range of patients with cancer at risk or with malnutrition,^43^ while others have shown improvements in nutritional status and unplanned admissions.^44^ Heterogeneity in nutrition intervention design, consultation frequency, and timing (ie, pre-surgery, post-surgery, or during chemoradiotherapy) for a range of cancer diagnoses, likely explains the mixed findings on body weight from reviews. On the whole, the literature supports that dietary counseling improves nutritional status and body weight.^11,12^ However, whether muscle mass along with body weight can be maintained through individualized dietary counseling across the cancer treatment continuum, requires further investigation.

**Table 1.** Summary of nutrition, exercise, or multimodal RCTs effects on weight and body composition

Author (year), country	Participant characteristics (n, treatment type)	Cancer type	Intervention details for nutrition, exercise, and multimodal trials.	Comparator	Outcome measures (* denotes primary, if reported)	Results (body weight or composition only)
*Nutrition alone*						
Burden et al^15^ (2017) UK	101, post-surgery	Colorectal	*Dietary counseling with ONS* Single dietary counseling at baseline aimed to increase energy and protein. *ONS:* (10.1 KJ, 0.096 g protein per 250 mL) commenced once the surgery date was confirmed. Pre-operative, 5 ONS provided, and post-operatively daily ONS was provided.	*Dietary counseling* Single dietary counseling post-operatively with ONS bottles of water.	Infection *, complications, length of stay in hospital, body weight	There was a significant difference in the percentage of weight lost between groups both pre-operatively (intervention 4.1% IQR 1.7–7.0 vs controls 6.7% IQR 2.6–10, *P* = .021) and post-operatively (intervention 7.4% IQR 4.3–10 vs controls 10.2% IQR 5.1–18.5, *P* = 0.016).
Cereda et al^16^ (2018) Italy	159, scheduled for radiotherapy	Head and neck	*Dietary counseling with ONS* Individualized weekly face-to-face dietary counseling with a dietitian commenced at the start of radiotherapy to meet energy requirements (Harris-Benedict derived), protein (1.2 g/kg^−1^), symptom management. *ONS*: Twice/day providing 500 kcal, 23 g protein, 1.9 g omega n−3.	*Dietary counseling* Dietary counseling as described above. ONS was provided if < 60% estimated requirements were not met.	Body weight *, dietary intake, muscle strength, quality of life, and treatment tolerance.	Significant between-group mean difference in body weight in favor of the ONS group (+ 1.6 kg, 95%CI 0.5-2.7, *P* = .006)
Cereda et al^17^ (2019) Italy	166, chemotherapy, diagnosed with malnutrition	Mixed, advanced cancer	*Dietary counseling with whey protein ONS* Individualized weekly-to-monthly face-to-face dietary counseling with a dietitian to meet energy requirements (Harris-Benedict-derived), protein (1.5 g/kg^−1^), symptom management. EN used if <60% requirements across 2 weeks after ONS trialed. *ONS:* Whey protein twice/day of 20 g protein (cysteine-rich, lactose-free) mixed in water or milk.	*Dietary counseling* Individualized weekly-to-monthly face-to-face dietary counseling with a dietitian to meet energy requirements (Harris-Benedict-derived), Protein (1.2 g/kg^−1^), symptom management. ONS twice/day providing 300-600 kcal, 20-40g protein, when < 60% estimated energy requirements across two consecutive weeks. EN used if <60% requirements across 2 weeks after ONS trialed.	Phase angle *, body weight, composition, hand grip strength, treatment toxicity	Significant between-group difference in body weight (MD 1.7, 95%CI 0.2, 3.1, *P* = .023) and fat-free mass (MD 0.46, 95%CI 0.02, 0.90, *P* = .041) at the end of the study (3-months).
Chen et al^18^ (2023) China	78, chemotherapy	Gastrointestinal	*Dietary counseling with ONS* Escalating decision-making on nutrition through (1) dietary counseling to meet energy (25-30 kcal/kg^−1^) and protein (1.2 g/kg^−1^), (2) counseling with ONS, (3) enteral nutrition, (4) partial enteral and parenteral nutrition, (5) full parenteral nutrition. Escalated each step based on failing to meet < 60% energy requirements in 3-5 days. Intervention commenced at chemotherapy and followed up weekly.	Usual care which included simple nutrition assessment and education.	PG-SGA *, body weight, hand grip strength, triceps skin fold, arm muscle circumference, calf circumference, quality of life.	Significant between-group difference in weight (MD 4.2 ± 1.9 kg vs −2.6 ± 1.7 kg).
Meng et al^19^ (2021) China	333, post-surgery and at risk of malnutrition	Gastric	*Dietary counseling with ONS* Fortnightly dietary counseling to increase energy and protein intake (unknown requirements). *ONS*: 100 kcal and 4.1g protein per 100 mL (goal of 500 mL).	*Dietary counseling* As described in the intervention arm. ONS provided if ≥5% weight loss is observed.	Weight, skeletal muscle index, sarcopenia, treatment tolerance, quality of life.	Significant between-group difference in weight loss (estimated −2.1 kg vs −4 kg) and skeletal muscle index at (estimated 40 cm^2^/m^2^ vs 38 cm^2^/m^2^) 3 months (post-program)
Molassiotis et al^20^ (2021) Australia & Hong Kong	74 patients and 54 family care givers, stage III-IV cancer with a life expectancy of ≥6 months and at risk of malnutrition.	Mixed	*Family-centered nutrition intervention* Three structured sessions with a dietitian over 4 weeks through telehealth with family/carers to individually meet energy and protein requirements (unknown) and considering symptom management. ONS may have been recommended.	Usual care nutrition advice and symptom management through palliative care team.	Feasibility *, quality of life, PG-SGA, eating-related distress, weight.	No significant differences between groups in weight
Patursson et al^21^ (2021) Svabosgøta	30, outpatient radiotherapy to the gastrointestinal and pelvic area	Mixed	*Dietary counseling with ONS* Individualized dietary counseling from a dietitian to meet nutritional requirements (unknown). *ONS*: 33.8 g protein, 2.2 g EPA, 1.1 g DHA, and 2500 kJ/day.	Usual care nutrition education provided by nurses	Body weight *, dietary intake, quality of life.	No significant difference between-group differences in weight
Tan et al^22^ (2021) China	212, post-surgery and at risk of malnutrition	Colorectal	*Dietary counseling with ONS* Fortnightly dietary counseling to increase energy and protein intake (unknown requirements). *ONS*: 100 kcal and 4.1g protein per 100 mL (goal of 500 mL).	*Dietary counseling* As described in the intervention arm. ONS provided if ≥5% weight loss is observed.	Body weight, skeletal muscle index, sarcopenia, treatment tolerance, quality of life.	No significant between-group differences in weight post-program, however significant increase in skeletal muscle index compared to the control group at 3-months (estimate mean 40 cm^2^/m^2^ vs 38 cm^2^/m^2^)
Zhu et al^23^ (2019) China	104, post-surgery	Gastrointestinal	*Dietary counseling with ONS* Dietary guidance from treating doctor (requirements unknown). *ONS*: 500 kcal, ~70 g protein per day for 90 days post discharge.	*Dietary counseling* The same dietary guidance from treating doctor.	Body weight *, biochemical markers, infection and complications, quality of life.	Significant between-group difference in weight at 60 days (1.34 ± 0.53 kg vs −1.01 ± 0.54 kg) and 90 days (1.35 ± 0.73 kg vs −1.59 ± 0.81 kg)
*Exercise alone*						
Anandavadivelan et al^24^ (2022) Sweden	161, 1-year post-surgery	Esophageal	*Exercise prescription*: 12 weeks, home-based, weekly telephone calls. Education regarding PA and 5 UL and LL strength exercises with resistance bands and body weight, 10 repts × 2 sets. *F*: ×2/week. *I:* NR, progressed as acceptable to the patient. *T*: NR.	Education regarding PA guidelines for older people.	Muscle strength* (handgrip and 30secSTS) and muscle mass* (bio-impedance)	No significant between-group changes in muscle mass (intervention MD 0.14 (−1.34 to 1.61) kg and control group 0.33 (−0.83 to 1.48) kg, *P* = .84
Arrieta et al^25^ (2019) France	301, at commencement of curative treatment, aged ≥ 70 years	Lymphoma or carcinoma (36% breast)	*Exercise prescription:* Unsupervised, home-based. Phone calls ×2/month for 6 months and monthly from months 6-12. Aerobic, strength (UL and LL), flexibility, proprioception, and balance. *F*: ×2 days/week. *I*: low to high. RT—×10 reps, load added as tolerated) *T*: NR	Usual care booklet	Physical function (Short Physical Performance Battery)*, physical activity, cognition, hospitalization/institutionalization, falls, mortality at 12 months	Muscle mass not assessed.
Cheng et al^26^ (2021) China	120, during chemo +/- radiotherapy	Lung, gastric, or breast cancer	*Exercise prescription (3-arm trial):* *Arm 1:* Tai Chi 12 weeks *F*: ×3/week *I*: high-intensity (60% 1RM) *T*: 40 minutes/session *Arm 2:* Resistance exercise (6 exercises for UL and LL with weights or body weight) 12 weeks *F*: ×3/week *I*: low-intensity (30% 1RM) *T*: 10 sets × 3 minutes each exercise	Usual care	Muscle strength (1RM), weight, body composition (fat mass, lean body mass), fatigue, health-related quality of life, anxiety, depression, sleep quality and adverse events	Body weight increased in both resistance groups compared to the control group (*P* < 0.05). LM increased and FM decreased significantly in the high-intensity group compared to the low-intensity of Tai Chi groups (*P* < .05)
Grote et al^27^ (2018) Germany	20, during inpatient or outpatient RTx (65% combined chemoRTx), stage I-IV	HNC	*Exercise prescription:* Supervised during RTx (mean 16.6, range 13-25 sessions). Resistance (machine weights—leg press, lat pull-downs, and chest press), 8-12 reps × 3 sets. Progressed if RPE < 7 by increasing weight (UL 2.5 kg and LL 5kg). *F*: x 3/week. *T*: 30 minutes/session	Usual care (no muscle strengthening technique but could include IP physio)	Feasibility*, fatigue, health-related quality of life, physical performance (6MWT), muscle strength (elbow flexion, knee extension), body composition (BIA)	Baseline (T1), after 7 weeks of RTx (T1) and 8 weeks after RTx2 (T3). Fat mass—decreased by 20% UC and 30% IG (T1-T3), decreased by 2% UC and 16% IG (T2-T3), *P* = .545. Lean mass—decreased by 3% UC and increased by 1% IG (T1-T3), *P* = .267
Hacker et al^28^ (2017) USA	75, post allogeneic or autologous stem cell transplant	Hematological	*Exercise prescription:* AROM ×2/week during IP admission. Following discharge ×6 weeks of RT (11 exercises—UL, LL, abdominals with resistance bands and body weight). Progressed by increasing repetitions, then sets, then resistance. F: ×3/week (one-on-one supervised ×1-2/week, ×1-2 unsupervised at home). *I*: Borg 13 (moderate) *T*: NR	During IP admission—2 visits/week to discuss hospital experience. Following discharge 6-week health education program. *F*: ×1/week (one-on-one education).	Physical activity (objective* and patient-reported), fatigue, muscle mass (rectus femoris muscle ultrasound CSA), muscle strength (handgrip, arm curl test), functional ability (timed stair climb, TUG test, 15-ft walk time and 30 seconds CST), HRQoL	Pre-transplant to post-intervention measures. Non-significant between-group differences for muscle mass (*P* > .05)
Kamel et al^29^ (2020) Egypt	40, resectable and non-resectable, stage I-IV	Pancreatic	*Exercise prescription:* 12-weeks, supervised. Resistance (machine-based, 8 exercises for UL and LL). *F*: ×2 sessions/week. *I*: commenced 5 exercises, 20 reps × 1-2 sets 50%-60% 1RM, progressed week 5 to 8 exercises 8-12 reps × 3 sets 60%-80% 1RM. T: 60 minutes/session	No exercise, physio phone contact once/month about negative cancer therapy outcomes plus nutritional and psychosocial support	Mobility, muscle strength, and lean body mass	Pre–post intervention measures. Improvements in UL and LL lean mass (6.28-6.46 vs 6.31-6.23 kg and 16.31-16.58 vs 16.4-16.31 kg, respectively, favoring IG)
Lavigne et al^30^ (2020) Canada	22, completed RT +/- chemo (stage I-IV). Surgery (68%) and concomitant chemo (82%).	HNC	*Exercise prescription:* 12 weeks, supervised, novel strength training—eccentrically overloaded training (squats, intensity “to tolerance”) and NMES contractions (66 bilateral, 40 Hz, pulse duration 180, 5 s contraction, 10 s rest, 1.5 s ramp-up, 0.75 s ramp-down). *F*: ×3/week, 2 sets of 8 reps. *I*: NR *T*: NR.	Conventional strength exercises 5 exercises using body weight or dumbbells (squats, lunge, step up, knee extension) 2 sets of 8. intensity “to tolerance.”	Feasibility* (recruitment, completion, adherence, progression). HRQoL, fatigue, thigh muscle CSA ultrasound, neuromuscular function (isometric dynamometry and femoral nerve electrical stimulation), 30 seconds STS	Pre-post whole group: KE force increased by 22 (23)% *P* < .001, mass: 19% (23) increase in VL CSA *P* = .004, 17 (22)% increase in RF CSA *P* = .006)
Rosenberger et al^31^ Germany	25, TKI-based anti-tumor therapy	Mixed	*Exercise prescription:* 12-week, supervised, resistance training—8 machine-based exercises of major muscle groups (2 sets × 12 reps at 12RM). Progressed by weight increase. *F*: x2/week. *I:* NR *T*: 60 minutessss/session.	No intervention.	Feasibility*, fatigue, HRQoL, depression, muscle strength, cardiorespiratory fitness (CPET), anthropometric measures.	Pre–post-intervention measures. Unadjusted model significant change in body weight favoring IG 1.2 kg (−0.3 to 2.8) vs UC −0.9 kg (−2.6 to 0.7), *P* = .042 (adjusted for baseline *P* = .05)
Scott et al^32^ (2021) USA	90, < 1 or ≥ 10 years post definitive Rx completion (Stage I-IIIB or LS)	Lung	*Exercise prescription:* 16 weeks of individual, supervised sessions. 3 groups: AT (aerobic, interval training—stationary cycle ergometry), RT (resistance training with weights—14 UL and LL exercises, 6-18 reps × 3 sets), CT (aerobic and resistance combined). *F*: ×3/week. *I*: commenced 50%-60% 1RM and progressively increased to 75%-85% 1RM. *T*: 30-60 minutes/session.	Attention control—stretching exercises, 20-45 minutes/session.	Cardiorespiratory fitness*, maximal muscle strength, weight, percentage of lean and fat mass (DEXA or air displacement plethysmography), HRQoL, pain, sleep quality, and safety.	RT was associated with near-significant improvements in lean mass (1.6% (−0.2 to 3.4) *P* = .08) and reductions in fat mass (−1.6% (−3.4 to 0.2) *P* = .08) and no difference in weight (0.6 kg (−0.6 to 1.7) *P* = .33), compared with AC
Wiskemann et al^33^ Germany	65, resectable or non-resectable (stage I-IV). Surgery + adjuvant chemo (83%)	Pancreatic	*Exercise prescription:* 6 months. Two groups either: RT1 supervised (living < 20 km from study center) or RT2 home-based with weekly phone calls. UL and LL strength training with weights. *F*: ×2/week. *I*: RT1 60%-80% 1RM and RT2 14-16 Borg RPE. *T*: 60 minutes/session	Usual care, patient calls ×1/month regarding side effects and advised not to change exercise behavior.	Adherence, feasibility*, muscle strength, cardiorespiratory fitness, body weight	No significant changes in body weight from baseline (RT1 3.2 (3.7)%, RT2 −0.4 (6.2)%, control 0.8 (5.8)%.
Wochner et al^34^ Germany (Secondary analysis of Wiskemann 2019)					Body composition: CT scans (L3/4) fat area (total, visceral, subcutaneous, intramuscular) and visceral-subcutaneous fat ratio, muscle area, muscle density, and SMI. Overall survival.	Pre-post—no significant effects on body composition (*P* > .05). Loss of muscle mass was a predictor of poor OS (visceral fat ratio HR = 2.084, *P* = .014)
Yen et al^35^ Taiwan	84, during chemotherapy, stage NR	HNC	*Exercise prescription:* 8-week. Aerobic and resistance (10 UL and LL exercises with resistance band, 10 reps, 1-3 sets. *F*: ×3 + days/week. *I*: 60%-70% HR max; RT “somewhat heavy”-“heavy” RPE. *T*: 40-50 minutes/session.	NR	Body composition, functional exercise capacity (6MWT), pre and post 6MWT HR, BP, and rate-pressure product	Pre-post-intervention measures. Body weight (kg) significantly reduced in the control group (mean (SD) 59.1 (11.3) vs 58.1 (11.4)). BMI (kg/m^2^) significantly reduced in the control group (mean (SD) 22.3 (3.8) vs 21.9 (4)). Visceral fat was significantly reduced in the intervention group (mean (SD) 7.9 (4.7) vs 7.4 (4.5)). Skeletal muscle rate significantly increased in the intervention group (mean (SD) 32.1 (3.8) vs 33.6 (4.1)).
*Multimodal interventions*						
Gillis et al^36^ (2019) Canada	76, pre- or post-colorectal surgery.	Colorectal	*Prehab* Duration 4 weeks prior to surgery to 8-weeks post-surgery. *Exercise* Aerobic and resistance training that is home-based. *F*: 3/7. *I*: NR *T*: 50 minutes per session. Plus weekly supervised group exercise. *Nutrition* Individualized dietary counseling from a dietitian (1.2 g/IBW/day) focused on symptom control, and optimizing body composition, plus whey protein supplement (nutritional breakdown not provided). *Rehab* Identical to above but baseline consultation occurred a few days prior to surgery, and the intervention started post-surgery. *Other* Anxiety-reduction consultation with a psychologist weekly.	NA	Body composition * 6MWT Feasibility (intervention compliance)	Significant increase in lean mass (change from baseline =+0.4 kg; 95% CI: 0.03-0.7 to kg, *P* = .031) and lost FM (change from baseline =−0.6 kg, 95%CI: −1.0 to −0.2 kg, *P* = .005) in the pre-surgery period. Baseline to 8 weeks shows no changes in lean mass. Rehabilitation group showed a reduction in lean mass (change baseline to 8 weeks: −1.1 kg, 95%CI: −2.3 to −0.02 kg, *P* = .045).
Hall et al.^37^ (2021) UK	45, diagnosed with incurable cancer with predicted survival > 3 months.	Mixed	*Exercise* Weekly consults with an exercise physiologist for home-based aerobic exercise (*F*: NR, *D*: 60 minutes/week, *I*: moderate, Borg scale 3-4) and resistance training of body weight exercises targeting major muscle groups. *Nutrition* Weekly consults with a dietitian for individualized counseling on food preferences to meet requirements plus ONS (330 kcal, 1 g EPA *OR* oral capsules of 2 g EPA).	NA	Feasibility*, quality of life, physical activity, and body weight.	Non-significant between-group differences in body mass post-program (median with IQR) Intervention +1 kg, (−2 to 2), control −3 kg (−2, 0).
Mikkelsen et al^38^ (2022) Denmark	84, during or following systemic treatment, 93% metastatic disease	Lung, pancreatic, biliary tract	*Exercise* 12 weeks, supervised (resistance) and home-based (walking with a pedometer). Resistance (machine-based weights, 7 exercises for UL and LL) and stretching. *F*: ×2/week supervised and daily home-based. I: commenced at 15 RM × 2 sets and progressed to 10RM × 3 sets *T*: 60 minutes/session *Nutrition* Dietary counseling bi-weekly to achieve 200-300 calories, 12-18 g protein *Other* Two sessions of health education from nurses	Usual care (not asked to refrain from exercise)	Physical function (patient-reported and performance-based (30 seconds STS*)), feasibility, physical capacity (6 MWT), handgrip strength, body composition (DEXA and BIA), symptom burden, health-related quality of life, mood, toxicities, hospital re-admissions	Pre-, mid-, and post-intervention. The significant difference in LM change of 0.9 kg (SE 0.4), *P* = .033 favoring IG.
Molenaar et al.^39^ (2022) International	251, operable, non-metastatic	Colorectal	Prehab *Exercise* 4-weeks, supervised. Aerobic (bicycle) and resistance (machines, body weight, bands for major muscle groups, 2 sets × 10 reps). *F*: x3 sessions/week. *I*: aerobic (HIIT—85%-90% peak power); resistance (65%-70% 1RM). *T*: 60 minutes/session *Nutrition* Education to balance macronutrients and achieve 1.5 g per kg of protein daily. *ONS*: 30 g whey protein supplement taken within 60 minutes of exercise. Multivitamin and Vitamin D supplements. *Other* Anxiety coping interventions—relaxation and deep breathing. Smoking cessation (if required)	Standard care ERAS pathway	30-day post-operative pulmonary complications*, 6MWT distance*, cardiorespiratory fitness, muscle strength (1RM and handgrip), nutritional status (PG-SGA), mental health (GAD-7 and PHQ-9), HRQoL (EORTC-QLQ-C30), hospital LOS, readmissions and mortality at 30 days.	Muscle mass not assessed. No significant differences in nutritional status (PG-SGA (median [IQR] post-intervention (pre-op) 0 [0,2] both groups).
O’Neill et al^40^ (2018) Ireland	43, post-operative (25 had neoadjuvant Rx and 9 adjuvant Rx)	Oesophageal	*Exercise* 12-week, supervised and unsupervised. Aerobic (commenced 30%-45% HRR and progressed weekly to 45-60% HRR) and resistance (2 sets 12 RM and progressed to 6 sets of 17RM) training. *F*: 2-3×/week. *I*: aerobic (start 30%-45% HRR and progress weekly to 45%-60% HRR). T: NR. *Nutrition* Dietary counseling and education on GI symptoms. Nutrition prescription NR.	Usual care (standard care as per best practice).	Cardiorespiratory fitness (CPET)*, body composition (anthropometrics and bioimpedance), HRQoL, PA (objective), adverse events.	Baseline, post-intervention, and 3 months post-intervention. No significant changes in body composition or anthropometrics at either timepoint apart from MUAC post-intervention *P* = .019 favoring IG (stable) vs decrease in UC.
Solheim et al^41^ (2017) Norway	46, during chemotherapy, stage III-IV	Lung and pancreatic	*Exercise* 6-week, home-based with weekly telephone contact for adherence. Aerobic (patient’s choice 30 minutes ×2/week) and resistance (6 UL and LL exercises with weights and body weight, 20 minutes) × 3/week. *I*: NR *Nutrition* Single nutritional counseling to increase meal frequency and energy intake. ONS 2× daily 220 mL of 1 g EPA. *Other*: Daily anti-inflammatories (celecoxib).	Standard care offered intervention after 6 weeks.	Feasibility*, weight, muscle mass (CT at L3), PA (objectively using accelerometry and 6MWT), handgrip strength, nutritional status, fatigue, safety and survival.	Pre-post intervention. Mean (SD) % change: weight 1.29 (3.41) vs −3.91 (3.67), *P* < .001 favoring IG; muscle mass −0.02 (0.07) vs −0.04 (0.06), *P* = .03.
Balstad et al^42^ (2020) (Secondary analysis of Solheim et al)					Estimates of sensitivity to change and between-group effect sizes for body weight, body composition, physical function, and metabolism.	Body weight ES large (1.2, *P* < .001), body composition ES small (range for different outcomes between 0.15 and 0.26, *P* > .05).

Abbreviations: AROM, active range of motion; BIA, bioelectrical impedance analysis; BP, blood pressure; CI, confidence interval; CPET, cardiopulmonary exercise testing; CT, computerized tomography; DHA, Docosahexaenoic acid; EPA, eicosapentaenoic acid; EN, enteral nutrition; ES, effect size; EORTC-QLQ, European Organization for Research and Treatment of Cancer Quality of Life Questionnaire; DEXA, dual-energy x-ray absorptiometry; F, frequency; GAD-7, general anxiety disorder; GI, gastrointestinal; HNC, head and neck cancer; HRQoL, health-related quality of life; HRR, heart-rate resting; I, intensity; IBW, ideal body weight; IQR, interquartile range; IG, intervention group; KE, kinetic energy; LL, lower limbs; LOS, length of stay; LM, lean mass; L3, lumbar vertebra 3; MUAC, mid-upper arm circumference; NA, not applicable; NMES, neuromuscular electrical stimulation; NR, not reported; ONS, oral nutrition supplement; PA, physical activity; PG-SGA, patient generated-subjective global assessment; PHQ-9, patient health questionnaire; UC, usual care; UL, upper limbs; RF CSA, rectus femoris cross-sectional area; RM, repetition maximum; RPE, rating of perceived exertion; RT, resistance training; RTx radiotherapy; SD, standard deviation; SGA, subjective global assessment; SMI, skeletal muscle index; STS, sit to stand test; T, time; TUG, timed up and go test; VL CSA, vastus lateralis cross-sectional area; 6MWT, 6-minute walk test.

Oral nutrition supplements (ONS) are recommended if oral intake is inadequate to meet estimated energy and protein requirements to maintain weight and muscle mass with continued dietary counseling.^45^ A meta-analysis of 12 RCTs evaluating the effects of dietary counseling with ONS (protein with or without omega-3) on malnutrition found body weight was maintained (MD + 1.31 kg; *P* = .02) during chemoradiotherapy.^46^ No benefit was found in terms of survival (*n* = 4), or treatment-related toxicity (*n* = 3), and there were inconsistent effects on muscle mass (*n* = 2).^46^ Another systematic review in head and neck cancers found ONS with dietary counseling maintain weight for up to 12 months post-treatment^47^; however, other clinical outcomes were not assessed. In 159 newly diagnosed patients with head and neck cancer, dietary counseling with ONS compared to dietary counseling only showed smaller reductions in body weight at the end of radiotherapy (67.7 vs 65.7 kg) and 3-months follow-up (68.3 vs 64.4 kg).^16^ The intervention arm reported less treatment change (radiotherapy dose-reduction) and improved completion (HR = 0.40; *P* = .029). Preventing unintentional weight loss through dietary counseling with ONS may improve treatment tolerance and completion and requires further investigation into other cancers and treatments. In addition, future trials must consider evaluating muscle mass given its relationship to chemotherapy-related toxicity and surgical complications to advance our understanding in this area.^48-50^

A large RCT (*n* = 506) evaluated an early, individualized dietary counseling or EN (if meeting < 75% estimated energy and protein requirements) intervention for malnourished patients during hospitalization, vs usual care, found reduced mortality (OR: 0.57; *P* = .027) in the intervention arm.^51^ Recent attention has turned to the economic benefit of nutrition intervention for people with cancer,^52^ and secondary analysis of the aforementioned RCT suggested that the intervention was cost-effective relative to usual care (CHF 43 711 vs 46 420 per patient).^53^ This emerging evidence of economic benefit calls for further international evaluations to reinforce the allocation of resources to dietetic services within cancer centers.

### Specialized oral nutrition supplements

Omega-3 long-chain polyunsaturated fatty acids, particularly eicosapentaenoic acid (EPA) and docosahexaenoic acid (DHA), offer anti-inflammatory properties which may counteract muscle loss and maintain body weight.^46,54^ A recent meta-analysis of 31 RCT’s evaluating ONS containing >600 mg of omega-3 consumed for >3 weeks compared to a control supplement showed no benefit on muscle mass (MD −0.47 kg; *P* = .59) or body weight (MD 4.02 kg; *P* = .06).^55^ However, adherence to the ONS was low. Participants in the control group were taking omega-3 supplements, and the majority of studies did not account for energy and protein intakes.^55^ Recently, β‐hydroxy β‐methylbutyrate (HMB), a compound supplemented in doses up to 3.0 g/day, has been used to prevent muscle deterioration by increasing muscle protein synthesis and decreasing protein breakdown.^56-58^ A systematic review evaluating HMB in ONS showed preliminary benefits for improving muscle mass, strength, and other clinical outcomes, however, improvements in body weight were not found.^58^ The additional benefits of omega-3 or HMB ONS, or a potential future combination of these with other nutrients, compounds, or ingredients,^59,60^ require further evaluation through well-designed, adequately powered RCT’s. These trials should be powered to detect clinically important differences in body weight and muscle mass, measure adherence to the ONS, and assess concurrent dietary intake.

### Enteral and parenteral nutrition support

There are limited randomized trials or reviews evaluating EN or PN during cancer treatment within the past 5 years and evidence directly evaluating changes in body weight or muscle mass within a hospital setting is scarce. These feeding approaches must be patient-centered and consider the psychosocial state, gastrointestinal function, nutrition impact symptoms, and the location and treatment of the tumor.^61,62^ Home EN improves the quality of life and reduces time spent in the hospital during cancer treatment. Meta-analysis of 4 RCTs shows that EN maintained BMI (WMD 0.70 kg; *P* = .02) and muscle mass (WMD 0.76 kg; *P* = .04) compared to normal oral intake after esophageal cancer surgery.^63^ Enteral nutrition supplemented with nutrients including omega-3, Vitamins A, D, or E, and amino acids arginine and glutamine, has potential benefits on immune function. A systematic review of 7 randomized trials evaluated if EN enhanced with omega-3, arginine, and/or glutamine, preserves weight, and potentially muscle mass.^64^ However only a few studies (*n* = 4) measured muscle mass. Two meta-analyses in gastrointestinal cancers found a reduction in postoperative complications from EN enhanced with omega-3, arginine, glutamine, and/or ribonucleic acid (RR = 0.29; *P* = .001) compared to standard EN,^65^ and showed reduced length of hospital stay (MD -−.03 days; *P* < .001) when compared to dietary counseling.^66^ However, changes in weight or muscle mass were not examined in the included studies highlighting a gap to be addressed in future trials.

## Exercise approaches to unintentional weight loss and loss of muscle mass

For people with cancer, the World Health Organization guidelines recommend 150-300 minutes of moderate or 75-150 minutes of vigorous, intensity aerobic exercise/week, and moderate intensity muscle strengthening activities ≥2 days/week (≥3 days/week in ≥65-year-olds) to achieve health benefits.^67^ In discussing the evidence for the role of exercise in managing unintentional weight loss and loss of muscle mass, interventions focusing on resistance exercise (either alone or combined with aerobic) are highlighted. Myofibrillar protein synthesis is stimulated by resistance training, making it more effective in achieving skeletal muscle hypertrophy than aerobic exercise.^68^ It is noted, however, that aerobic exercise plays an important role in preserving skeletal muscle mitochondrial function^69^ and may reduce circulating inflammatory markers such as C-reactive protein.^70^ Interventions are in progress (clincaltrials.gov NCT05307367) to provide a further understanding of the effects of resistance training on inflammatory markers and muscle mass in lung cancer.

Systematic review evidence of exercise-only interventions to prevent muscle loss, from the last 5 years, addressing ASCO, ESPEN, and COSA guideline future research areas is limited, interventions are heterogenous and effectiveness findings are mixed. During and following treatment non-significant between-group differences were reported in 3 systematic reviews.^71-73^ Meta-analysis of 34 resistance training trials during and after cancer treatment report increases in muscle mass favoring the intervention (MD 0.85 kg 0.26 to 1.43; *P* = .004).^74^ Details of relevant RCTs are summarized in Table 1 and include resistance-focused interventions during and following treatment in hematological,^28^ head and neck,^27,30,35^ esophageal,^24^ pancreatic,^29,33,34^ lung^32^ and mixed^25,26,31^ cancers. Resistance training interventions vary, but most include supervised sessions (alone or combined with unsupervised, home-based exercise), prescribe moderate-intensity concentric (muscle shortening) exercise, and range from 6 weeks^28^ to 6 months^33^ duration. Findings for anthropometry and body composition (weight, BMI, muscle mass, and fat mass) outcomes post-program are mixed with some RCTs reporting non-significant between-group differences,^24,27,28,32-34^ while others find significant effects favoring the intervention group.^26,29-31,35^ Differing modality effects, as highlighted in ESPEN guidelines, were investigated in a 4-arm RCT (aerobic, resistance, combined aerobic, and resistance and usual care) >1 year post definitive lung cancer treatment. Resistance exercise was superior to aerobic for body composition outcomes (aerobic vs resistance muscle mass MD (SD) (−2% (3), *P* = .04) and fat mass (−2% (3), *P* = .04)), with no difference in weight and no additional benefit of combined aerobic and resistance exercise.^32^ Greater effects on body composition were found for high compared to low-intensity Tai Chi (*P* < .05) in a mixed tumor group during chemotherapy.^26^ Longer-term effects of exercise interventions have not been investigated and further trials are required to determine the most effective resistance exercise type (eg, eccentric (muscle lengthening), used in one RCT,^30^ vs concentric) and exercise prescription needed to increase muscle mass.^75^ In broad terms, exercise interventions in cancer are reported in one study to be cost-effective.^76^ In the future, health economic evaluations should be run alongside RCTs to understand the cost-effectiveness of interventions specifically aimed at preventing muscle loss.

### Safety considerations

Individual patient assessment is critical to prescribing safe exercise in any patient, including those with a cancer diagnosis. Systematic review evidence from 65 trials supports a low risk (3.5%) of adverse events from exercise,^77^ with musculoskeletal injury or pain being the most common. Although few of these trials measured the impact on unintentional weight loss. In the presence of fever, infection, and chest pain exercise should not be undertaken. For patients with bony metastases care needs to be taken to avoid loading the malignant bone lesion site. Patients with cancer may fluctuate in their symptomology and exercise intensity and/or duration may need to be varied. More detailed criteria may be found in the Exercise and Sports Science Australia position statement^78^ and also the American College of Sports Medicine guidelines papers.^79^

## Multimodal approaches to unintentional weight loss/malnutrition

Multimodal interventions (combination of 2 or more therapies) are often used prior to cancer treatment (prehabilitation), during or after treatment (rehabilitation) where deconditioning and risk of malnutrition are heightened.^80^ Several systematic reviews show multimodal rehabilitation interventions can maintain weight and muscle mass through individualized dietary counseling, ONS, and structured resistance training.^81-84^ A meta-analysis shows multimodal interventions during cancer treatment showed no between-group differences in weight (MD 0.46 kg; *P* = .549) and muscle (MD 0.11 kg; *P* = .864).^81^ Other reviews show multimodal rehabilitation interventions are safe and maintain weight and muscle mass in a broad mix of cancers,^83^ yet varied effects were reported in upper gastrointestinal cancers specifically.^84^ Included studies in gastrointestinal cancer^84^ used a range of multimodal interventions; these were predominantly 1:1 dietary counseling, however, the exercise interventions varied between supervised and unsupervised home-based combined resistance and aerobic exercise which may explain the mixed findings.

Results from multimodal rehabilitation RCTs (Table 1) on weight and muscle mass are varied and limited to esophageal,^40^ and a mixed group of advanced cancers.^37,38^ An RCT (*n* = 84) showed supervised resistance training and home-based walking, with dietary counseling, compared to usual care, increased weight (1.4 kg vs 0.4 kg), and muscle mass (1.3 kg vs 0.4 kg) during systemic treatment for advanced cancer.^38^ While 12-week multimodal rehabilitation post esophageal surgery, showed no benefits to weight and muscle mass compared to the usual care group.^40^ Research interest has turned to the implementation of multimodal rehabilitation in health services. A pilot study showed that nurse-led malnutrition screening and referral to multidisciplinary treatment for patients with head and neck cancer increased the proportion of patients identified with malnutrition from 14% to 88% for appropriate management, avoided 3.92 unplanned admissions on average, and reduced healthcare expenditure by approximately $121K AU per annum.^85^ Evidence supports the use of multimodal interventions during or after cancer treatment to maintain or increase weight and muscle mass, and new models of care to prevent malnutrition and muscle loss and support the COSA position statement^13^ and add new evidence to the ASCO guidelines.^14^

Prehabilitation is a multidisciplinary bundle of care that identifies impairments and provides targeted interventions to improve patient outcomes^86-88^ and evidence has substantially grown in the last 5 years in oncology. Systematic reviews suggest that prehabilitation interventions maintain muscle mass following gastrointestinal cancer surgery,^87^ however, more broadly, weight and muscle mass are not typically reported in prehabilitation interventions.^89^ A large (*n* = 251) prehabilitation trial prior to colorectal cancer surgery, involving 4-week supervised inpatient high-intensity aerobic and resistance exercise, dietary counseling, ONS, and psychosocial support, showed no change in malnutrition (PG-SGA median (IQR) 1 (0-4), intervention vs 1 (0-3), control). While strength was increased post-operatively, weight and muscle mass were not assessed.^39^ Pooled analysis of 2 RCTs comparing multimodal prehabilitation to rehabilitation in patients with colorectal cancer scheduled for surgery showed an increase in muscle mass prior to surgery (estimated MD + 0.4 vs −0.1 kg), and attenuation in losses in muscle mass 4-weeks (−0.6 vs −1.5 kg) and 8-weeks (−0.1 kg vs −1.1 kg) post-surgery, relative to the rehabilitation-only group.^36^ Recent trials further support the current guidelines, and new evidence is needed in other cancer treatments (ie, chemoradiotherapy) and tumors where malnutrition risk is high to address ASCO guidelines and future research recommendations.

## Conclusion and future directions

The evidence for managing, treating, and preventing unintentional weight and muscle loss, and malnutrition is strong for nutrition, emerging for multimodal interventions, and limited and for exercise-only trials. Dietary counseling with ONS shows benefits for maintaining weight in cancers and treatments where malnutrition risk is high. Protein and omega-3 ONS, with or without dietary counseling may mitigate muscle loss during cancer treatment, but the evidence is low and across the nutrition literature body composition is rarely measured. The optimal dose of protein (ie, type of protein, amino acid profile, and timing) and other dietary ingredients/nutrients (ie, HMB) effects on muscle mass require further investigation, and several trials are underway.^90^ Exercise, particularly resistance training, may offer great benefits in preserving and building muscle when weight loss is present during cancer treatment. However, further research identifying the optimal prescription (ie, frequency, intensity, type, and time) for achieving muscle hypertrophy is needed for patients at risk or with malnutrition. Multimodal trials prevent weight and muscle loss across multiple cancer types and treatments. Prehabilitation interventions potentially offset some or all of the unintentional weight and muscle loss developed after cancer surgery, however, changes in body composition and malnutrition have not been the primary outcome and are often only reported at baseline. Lastly, models of care that involve validated screening, assessment, and multimodal interventions for malnutrition in new treatments (eg, immunotherapy and targeted treatments) and new delivery modes such as online are needed to reflect recent changes in practice.

## Data Availability

No new data were generated or analysed in support of this research.

## References

[1] Marshall KM , Loeliger J, Nolte L, Kelaart A, Kiss NK. Prevalence of malnutrition and impact on clinical outcomes in cancer services: a comparison of two time points. Clin Nutr. 2019;38:644-651. 10.1016/j.clnu.2018.04.007 29789167

[2] Pressoir M , Desné S, Berchery D, et al. Prevalence, risk factors and clinical implications of malnutrition in French comprehensive cancer centres. Br J Cancer. 2010;102:966-971. 10.1038/sj.bjc.6605578 20160725 PMC2844030

[3] Cederholm T , Jensen GL, Correia MITD, et al; GLIM Core Leadership Committee, GLIM Working Group. GLIM criteria for the diagnosis of malnutrition—a consensus report from the global clinical nutrition community. J Cachexia Sarcopenia Muscle. 2019;10:207-217. 10.1002/jcsm.12383 30920778 PMC6438340

[4] Compher C , Cederholm T, Correia MITD, et al. Guidance for assessment of the muscle mass phenotypic criterion for the Global Leadership Initiative on Malnutrition diagnosis of malnutrition. JPEN J Parenter Enteral Nutr. 2022;46:1232-1242. 10.1002/jpen.2366 35437785

[5] Hanna L , Nguo K, Furness K, Porter J, Huggins CE. Association between skeletal muscle mass and quality of life in adults with cancer: a systematic review and meta‐analysis. J Cachexia Sarcopenia Muscle. 2022;13:839-857. 10.1002/jcsm.12928 35156342 PMC8977976

[6] Marín Caro MM , Laviano A, Pichard C. Impact of nutrition on quality of life during cancer. Curr Opin Clin Nutr Metab Care. 2007;10:480-487. 10.1097/MCO.0b013e3281e2c983 17563467

[7] Lis CG , Gupta D, Lammersfeld CA, Markman M, Vashi PG. Role of nutritional status in predicting quality of life outcomes in cancer—a systematic review of the epidemiological literature. Nutr J. 2012;11:27. 10.1186/1475-2891-11-27 22531478 PMC3408376

[8] Findlay M , White K, Brown C, Bauer JD. Nutritional status and skeletal muscle status in patients with head and neck cancer: impact on outcomes. J Cachexia Sarcopenia Muscle. 2021;12:2187-2198. 10.1002/jcsm.12829 34676673 PMC8718020

[9] Vashi PG , Gorsuch K, Wan L, et al. Sarcopenia supersedes subjective global assessment as a predictor of survival in colorectal cancer. PLoS One. 2019;14:e0218761. 10.1371/journal.pone.0218761 31220163 PMC6586333

[10] Brown JC , Caan BJ, Meyerhardt JA, et al. The deterioration of muscle mass and radiodensity is prognostic of poor survival in stage I-III colorectal cancer: a population-based cohort study (C-SCANS). J Cachexia Sarcopenia Muscle. 2018;9:664-672. 10.1002/jcsm.12305 29766660 PMC6104108

[11] Arends J , Bachmann P, Baracos V, et al. ESPEN guidelines on nutrition in cancer patients. Clin Nutr 2017;36:11-48. 10.1016/j.clnu.2016.07.015 27637832

[12] Muscaritoli M , Arends J, Bachmann P, et al. ESPEN practical guideline: clinical Nutrition in cancer. Clin Nutr 2021;40:2898-2913. 10.1016/j.clnu.2021.02.005 33946039

[13] Kiss N , Loeliger J, Findlay M, et al. Clinical oncology society of Australia: position statement on cancer-related malnutrition and sarcopenia. Nutr Diet 2020;77:416-425. 10.1111/1747-0080.12631 32803904 PMC7540290

[14] Ligibel JA , Bohlke K, May AM, et al. Exercise, diet, and weight management during cancer treatment: ASCO guideline. J Clin Oncol. 2022;40:2491-2507. 10.1200/JCO.22.00687 35576506

[15] Burden ST , Gibson DJ, Lal S, et al. Pre-operative oral nutritional supplementation with dietary advice versus dietary advice alone in weight-losing patients with colorectal cancer: single-blind randomized controlled trial. J Cachexia Sarcopenia Muscle. 2017;8:437-446. 10.1002/jcsm.12170 28052576 PMC5476846

[16] Cereda E , Cappello S, Colombo S, et al. Nutritional counseling with or without systematic use of oral nutritional supplements in head and neck cancer patients undergoing radiotherapy. Radiother Oncol. 2018;126:81-88. 10.1016/j.radonc.2017.10.015 29111172

[17] Cereda E , Turri A, Klersy C, et al. Whey protein isolate supplementation improves body composition, muscle strength, and treatment tolerance in malnourished advanced cancer patients undergoing chemotherapy. Cancer Med. 2019;8:6923-6932. 10.1002/cam4.2517 31568698 PMC6853834

[18] Chen L , Zhao M, Tan L, Zhang Y. Effects of five-step nutritional interventions conducted by a multidisciplinary care team on gastroenteric cancer patients undergoing chemotherapy: a randomized clinical trial. Nutr Cancer. 2023;75:197-206. 10.1080/01635581.2022.2104329 35903847

[19] Meng Q , Tan S, Jiang Y, et al. Post-discharge oral nutritional supplements with dietary advice in patients at nutritional risk after surgery for gastric cancer: a randomized clinical trial. Clin Nutr. 2021;40:40-46. 10.1016/j.clnu.2020.04.043 32563598

[20] Molassiotis A , Brown T, Cheng HL, et al. The effects of a family-centered psychosocial-based nutrition intervention in patients with advanced cancer: the PiCNIC2 pilot randomised controlled trial. Nutr J. 2021;20:2. 10.1186/s12937-020-00657-2 33388075 PMC7778804

[21] Patursson P , Møller G, Muhic A, Andersen JR. N-3 fatty acid EPA supplementation in cancer patients receiving abdominal radiotherapy—a randomised controlled trial. Clin Nutr ESPEN. 2021;43:130-136. 10.1016/j.clnesp.2021.03.001 34024504

[22] Tan S , Meng Q, Jiang Y, et al. Impact of oral nutritional supplements in post-discharge patients at nutritional risk following colorectal cancer surgery: a randomised clinical trial. Clin Nutr. 2021;40:47-53. 10.1016/j.clnu.2020.05.038 32563599

[23] Zhu MW , Yang X, Xiu DR, et al. Effect of oral nutritional supplementation on the post-discharge nutritional status and quality of life of gastrointestinal cancer patients after surgery: a multi-center study. Asia Pac J Clin Nutr. 2019;28:450-456. 10.6133/apjcn.201909_28(3).0004 31464391

[24] Anandavadivelan P , Malberg K, Vikstrom K, et al. Home-based physical activity after treatment for esophageal cancer—a randomized controlled trial. Cancer Med. 2023;12:3477-3487. 10.1002/cam4.5131 36812121 PMC9939163

[25] Arrieta H , Astrugue C, Regueme S, et al. Effects of a physical activity programme to prevent physical performance decline in onco-geriatric patients: a randomized multicentre trial. J Cachexia Sarcopenia Muscle. 2019;10:287-297. 10.1002/jcsm.12382 30829460 PMC6463460

[26] Cheng D , Wang X, Hu J, et al. Effect of Tai Chi and resistance training on cancer-related fatigue and quality of life in middle-aged and elderly cancer patients. Chin J Integr Med. 2021;27:265-272. 10.1007/s11655-021-3278-9 33420583

[27] Grote M , Maihöfer C, Weigl M, Davies-Knorr P, Belka C. Progressive resistance training in cachectic head and neck cancer patients undergoing radiotherapy: a randomized controlled pilot feasibility trial. Radiat. Oncol. 2018;13:215. 10.1186/s13014-018-1157-0 30400971 PMC6219249

[28] Hacker ED , Collins E, Park C, et al. Strength training to enhance early recovery after hematopoietic stem cell transplantation. Biol Blood Marrow Transplant. 2017;23:659-669. 10.1016/j.bbmt.2016.12.637 28042020

[29] Kamel FH , Basha MA, Alsharidah AS, Salama AB. Resistance training impact on mobility, muscle strength and lean mass in pancreatic cancer cachexia: a randomized controlled trial. Clin Rehabil. 2020;34:1391-1399. 10.1177/0269215520941912 32660264

[30] Lavigne C , Twomey R, Lau H, et al. Feasibility of eccentric overloading and neuromuscular electrical stimulation to improve muscle strength and muscle mass after treatment for head and neck cancer. J Cancer Surviv 2020;14:790-805. 10.1007/s11764-020-00893-9 32447575

[31] Rosenberger F , Wiskemann J, Vallet S, et al. Resistance training as supportive measure in advanced cancer patients undergoing TKI therapy-a controlled feasibility trial. Support Care Cancer 2017;25:3655-3664. 10.1007/s00520-017-3788-3 28667563

[32] Scott JM , Thomas SM, Herndon JE, et al. Effects and tolerability of exercise therapy modality on cardiorespiratory fitness in lung cancer: a randomized controlled trial. J Cachexia Sarcopenia Muscle. 2021;12:1456-1465. 10.1002/jcsm.12828 34658160 PMC8718021

[33] Wiskemann J , Clauss D, Tjaden C, et al. Progressive resistance training to impact physical fitness and body weight in pancreatic cancer patients: a randomized controlled trial. Pancreas. 2019;48:257-266. 10.1097/MPA.0000000000001221 30589829

[34] Wochner R , Clauss D, Nattenmüller J, et al. Impact of progressive resistance training on CT quantified muscle and adipose tissue compartments in pancreatic cancer patients. PLoS One. 2020;15:e0242785. 10.1371/journal.pone.0242785 33253318 PMC7703876

[35] Yen CJ , Hung CH, Kao CL, et al. Multimodal exercise ameliorates exercise responses and body composition in head and neck cancer patients receiving chemotherapy. Support Care Cancer 2019;27:4687-4695. 10.1007/s00520-019-04786-1 30949831

[36] Gillis C , Fenton TR, Sajobi TT, et al. Trimodal prehabilitation for colorectal surgery attenuates post-surgical losses in lean body mass: a pooled analysis of randomized controlled trials. Clin Nutr. 2019;38:1053-1060. 10.1016/j.clnu.2018.06.982 30025745

[37] Hall CC , Skipworth RJE, Blackwood H, et al. A randomized, feasibility trial of an exercise and nutrition-based rehabilitation programme (ENeRgy) in people with cancer. J Cachexia Sarcopenia Muscle. 2021;12:2034-2044. 10.1002/jcsm.12806 34612012 PMC8718057

[38] Mikkelsen MK , Lund CM, Vinther A, et al. Effects of a 12-week multimodal exercise intervention among older patients with advanced cancer: results from a randomized controlled trial. Oncologist. 2022;27:67-78. 10.1002/onco.13970 34498352 PMC8842365

[39] Molenaar CJL , Minnella EM, Coca-Martinez M, et al; PREHAB Study Group. Effect of multimodal prehabilitation on reducing postoperative complications and enhancing functional capacity following colorectal cancer surgery: the PREHAB randomized clinical trial. JAMA Surg. 2023;158:572-581. 10.1001/jamasurg.2023.0198 36988937 PMC10061316

[40] O’Neill LM , Guinan E, Doyle SL, et al. The RESTORE randomized controlled trial: impact of a multidisciplinary rehabilitative program on cardiorespiratory fitness in esophagogastric cancer survivorship. Ann Surg. 2018;268:747-755. 10.1097/SLA.0000000000002895 30004915

[41] Solheim TS , Laird BJA, Balstad TR, et al. A randomized phase II feasibility trial of a multimodal intervention for the management of cachexia in lung and pancreatic cancer. J Cachexia Sarcopenia Muscle. 2017;8:778-788. 10.1002/jcsm.12201 28614627 PMC5659068

[42] Balstad TR , Brunelli C, Pettersen CH, et al. Power comparisons and clinical meaning of outcome measures in assessing treatment effect in cancer cachexia: secondary analysis from a randomized pilot multimodal intervention trial. Front Nutr. 2020;7:602775. 10.3389/fnut.2020.602775 33585533 PMC7874039

[43] Zhang F , Jin Y, Qiang W. The effects of dietary advice on malnutrition in cancer patients: a systematic review and meta-analysis. Support Care Cancer. 2020;28:1579-1585. 10.1007/s00520-019-05222-0 31836940

[44] Tunzi L , Funk T, Brown T, Findlay M, Bauer J. Optimal frequency of individualised nutrition counselling in patients with head and neck cancer receiving radiotherapy: a systematic review. J Hum Nutr Diet 2022;35:223-233. 10.1111/jhn.12919 34003532

[45] Denlinger CS , Sanft T, Moslehi JJ, et al. NCCN Guidelines Insights: survivorship, Version 2.2020: featured Updates to the NCCN Guidelines. J Natl Compr Canc Netw. 2020;18:1016-1023. 10.6004/jnccn.2020.0037 32755975 PMC7785060

[46] de van der Schueren ME , Laviano A, Blanchard H, et al. Systematic review and meta-analysis of the evidence for oral nutritional intervention on nutritional and clinical outcomes during chemo(radio)therapy: current evidence and guidance for design of future trials. Ann. Oncol. 2018;29:1141-1153. 10.1093/annonc/mdy114 29788170 PMC5961292

[47] Leis C , Arthur AE, Chen X, Greene MW, Frugé AD. Systematic review of nutrition interventions to improve short term outcomes in head and neck cancer patients. Cancers. 2023;15:822. 10.3390/cancers15030822 36765780 PMC9913111

[48] Kurk S , Peeters P, Stellato R, et al. Skeletal muscle mass loss and dose-limiting toxicities in metastatic colorectal cancer patients. J Cachexia Sarcopenia Muscle. 2019;10:803-813. 10.1002/jcsm.12436 31094083 PMC6711417

[49] Pin F , Couch ME, Bonetto A. Preservation of muscle mass as a strategy to reduce the toxic effects of cancer chemotherapy on body composition. Curr Opin Support Palliat Care. 2018;12:420-426. 10.1097/SPC.0000000000000382 30124526 PMC6221433

[50] de Jong C , Chargi N, Herder GJM, et al. The association between skeletal muscle measures and chemotherapy‐induced toxicity in non‐small cell lung cancer patients. J Cachexia Sarcopenia Muscle. 2022;13:1554-1564. 10.1002/jcsm.12967 35301821 PMC9178405

[51] Bargetzi L , Brack C, Herrmann J, et al. Nutritional support during the hospital stay reduces mortality in patients with different types of cancers: secondary analysis of a prospective randomized trial. Ann. Oncol. 2021;32:1025-1033. 10.1016/j.annonc.2021.05.793 34022376

[52] Parsons HM , Forte ML, Abdi HI, et al. Cost-Effectiveness of Nutrition Interventions. In: Nutrition as Prevention for Improved Cancer Health Outcomes [Internet]. Agency for Healthcare Research and Quality (US); 2023. Accessed June 23, 2023. https://www.ncbi.nlm.nih.gov/books/NBK592519/37289928

[53] Schuetz P , Sulo S, Walzer S, Krenberger S, Brunton C. Nutritional support during the hospital stay is cost-effective for preventing adverse outcomes in patients with cancer. Front Oncol. 2022;12:916073. 10.3389/fonc.2022.916073 https://www.frontiersin.org/articles/10.3389/fonc.2022.916073. Accessed June 16, 2023. 36016618 PMC9396738

[54] de Aguiar Pastore Silva J , Emilia de Souza Fabre M, Waitzberg DL. Omega-3 supplements for patients in chemotherapy and/or radiotherapy: a systematic review. Clin Nutr. 2015;34:359-366. 10.1016/j.clnu.2014.11.005 25907586

[55] Lam CN , Watt AE, Isenring EA, de van der Schueren MAE, van der Meij BS. The effect of oral omega-3 polyunsaturated fatty acid supplementation on muscle maintenance and quality of life in patients with cancer: a systematic review and meta-analysis. Clin Nutr. 2021;40:3815-3826. 10.1016/j.clnu.2021.04.031 34130028

[56] Engelen MPKJ , Deutz NEP. Is β-hydroxy β-methylbutyrate an effective anabolic agent to improve outcome in older diseased populations? Curr Opin Clin Nutr Metab Care. 2018;21:207-213. 10.1097/MCO.0000000000000459 29406417 PMC5882564

[57] Holeček M. Beta-hydroxy-beta-methylbutyrate supplementation and skeletal muscle in healthy and muscle-wasting conditions. J Cachexia Sarcopenia Muscle. 2017;8:529-541. 10.1002/jcsm.12208 28493406 PMC5566641

[58] Prado CM , Orsso CE, Pereira SL, Atherton PJ, Deutz NEP. Effects of β‐hydroxy β‐methylbutyrate (HMB) supplementation on muscle mass, function, and other outcomes in patients with cancer: a systematic review. J Cachexia Sarcopenia Muscle. 2022;13:1623-1641. 10.1002/jcsm.12952 35301826 PMC9178154

[59] Prado CM , Purcell SA, Laviano A. Nutrition interventions to treat low muscle mass in cancer. J Cachexia Sarcopenia Muscle. 2020;11:366-380. 10.1002/jcsm.12525 31916411 PMC7113510

[60] Prado CM , Anker SD, Coats AJS, Laviano A, von Haehling S. Nutrition in the spotlight in cachexia, sarcopenia and muscle: avoiding the wildfire. J Cachexia Sarcopenia Muscle. 2021;12:3-8. 10.1002/jcsm.12673 33382196 PMC7890147

[61] Chow R , Bruera E, Chiu L, et al. Enteral and parenteral nutrition in cancer patients: a systematic review and meta-analysis. Ann Palliat Med. 2016;5:30-41. 10.3978/j.issn.2224-5820.2016.01.01 26841813

[62] Cotogni P. Enteral versus parenteral nutrition in cancer patients: evidences and controversies. Ann Palliat Med. 2016;5:42-49. 10.3978/j.issn.2224-5820.2016.01.05 26841814

[63] Zhang C , Hu LW, Qiang Y, et al. Home enteral nutrition for patients with esophageal cancer undergoing esophagectomy: a systematic review and meta-analysis. Front Nutr. 2022;9. https://www.frontiersin.org/articles/10.3389/fnut.2022.895422. Accessed July 4, 2023. 10.3389/fnut.2022.895422 PMC9366554 35967793

[64] Miller LJ , Douglas C, McCullough FS, Stanworth SJ, Calder PC. Impact of enteral immunonutrition on infectious complications and immune and inflammatory markers in cancer patients undergoing chemotherapy: a systematic review of randomised controlled trials. Clin Nutr. 2022;41:2135-2146. 10.1016/j.clnu.2022.07.039 36067585

[65] Cheng Y , Zhang J, Zhang L, Wu J, Zhan Z. Enteral immunonutrition versus enteral nutrition for gastric cancer patients undergoing a total gastrectomy: a systematic review and meta-analysis. BMC Gastroenterol. 2018;18:11. 10.1186/s12876-018-0741-y 29338698 PMC5771223

[66] Shen J , Dai S, Li Z, et al. Effect of enteral immunonutrition in patients undergoing surgery for gastrointestinal cancer: an updated systematic review and meta-analysis. Front Nutr. 2022;9:941975. 10.3389/fnut.2022.941975 35845793 PMC9277464

[67] Bull FC , Al-Ansari SS, Biddle S, et al. World Health Organization 2020 guidelines on physical activity and sedentary behaviour. Br J Sports Med. 2020;54:1451-1462. 10.1136/bjsports-2020-102955 33239350 PMC7719906

[68] Grgic J , Mcllvenna LC, Fyfe JJ, et al. Does aerobic training promote the same skeletal muscle hypertrophy as resistance training? a systematic review and meta-analysis. Sports Med 2019;49:233-254. 10.1007/s40279-018-1008-z 30341595

[69] Mijwel S , Cardinale DA, Norrbom J, et al. Exercise training during chemotherapy preserves skeletal muscle fiber area, capillarization, and mitochondrial content in patients with breast cancer. FASEB J. 2018;32:5495-5505. 10.1096/fj.201700968R 29750574

[70] Mavropalias G , Sim M, Taaffe DR, et al. Exercise medicine for cancer cachexia: targeted exercise to counteract mechanisms and treatment side effects. J Cancer Res Clin Oncol. 2022;148:1389-1406. 10.1007/s00432-022-03927-0 35088134 PMC9114058

[71] Lee J. The effects of resistance training on muscular strength and hypertrophy in elderly cancer patients: a systematic review and meta-analysis. J Sport Health Sci. 2022;11:194-201. 10.1016/j.jshs.2021.02.002 33592324 PMC9068528

[72] Liu X , Xu X, Cheung DST, et al. The effects of exercise with or without dietary advice on muscle mass, muscle strength, and physical functioning among older cancer survivors: a meta-analysis of randomized controlled trials. J Cancer Surviv Res Pract. 2023;18(5):1-9. 10.1007/s11764-023-01396-z PMC10236397 37266818

[73] Grande AJ , Silva V, Sawaris Neto L, et al. Exercise for cancer cachexia in adults. Cochrane Database Syst Rev. 2021;3:CD010804. 10.1002/14651858.CD010804.pub3 33735441 PMC8094916

[74] Koeppel M , Mathis K, Schmitz KH, Wiskemann J. Muscle hypertrophy in cancer patients and survivors via strength training. A meta-analysis and meta-regression. Crit Rev Oncol Hematol. 2021;163:103371. 10.1016/j.critrevonc.2021.103371 34062243

[75] Bettariga F , Bishop C, Taaffe DR, et al. Time to consider the potential role of alternative resistance training methods in cancer management? J Sport Health Sci. 2023;12:715-725. 10.1016/j.jshs.2023.06.007 37399886 PMC10658316

[76] Wang Y , McCarthy AL, Hayes SC, et al. Economic evaluation of exercise interventions for individuals with cancer: a systematic review. Prev Med. 2023;172:107491. 10.1016/j.ypmed.2023.107491 36965520

[77] Fuller JT , Hartland MC, Maloney LT, Davison K. Therapeutic effects of aerobic and resistance exercises for cancer survivors: a systematic review of meta-analyses of clinical trials. Br J Sports Med. 2018;52:1311. 10.1136/bjsports-2017-098285 29549149

[78] Hayes SC , Newton RU, Spence RR, Galvão DA. The Exercise and Sports Science Australia position statement: exercise medicine in cancer management. J Sci Med Sport. 2019;22:1175-1199. 10.1016/j.jsams.2019.05.003 31277921

[79] Campbell KL , Winters-Stone K, Wiskemann J, et al. Exercise Guidelines for Cancer Survivors: consensus statement from International Multidisciplinary Roundtable. Med Sci Sports Exerc. 2019;51:2375-2390. 10.1249/MSS.0000000000002116 31626055 PMC8576825

[80] Merchant Z , Denehy L, Santa Mina D, Alibhai S, Moore J. Prehabilitation and rehabilitation in older adults with cancer and frailty. In: Gomes F, ed. Frailty in Older Adults with Cancer. Springer International Publishing; 2022:155-176. 10.1007/978-3-030-89162-6_9

[81] Baguley BJ , Dalla Via J, Fraser SF, Daly RM, Kiss N. Effectiveness of combined nutrition and exercise interventions on body weight, lean mass, and fat mass in adults diagnosed with cancer: a systematic review and meta-analysis. Nutr Rev. 2022;81:625-646. 10.1093/nutrit/nuac079 36206176

[82] Hall CC , Cook J, Maddocks M, et al. Combined exercise and nutritional rehabilitation in outpatients with incurable cancer: a systematic review. Support Care Cancer 2019;27:2371-2384. 10.1007/s00520-019-04749-6 30944994 PMC6541700

[83] Schmidt T , Süß P, Schulte DM, Letsch A, Jensen W. Supportive care in oncology—from physical activity to nutrition. Nutrients. 2022;14:1149. 10.3390/nu14061149 35334806 PMC8954702

[84] Sadeghi F , Mockler D, Guinan EM, Hussey J, Doyle SL. The effectiveness of nutrition interventions combined with exercise in upper gastrointestinal cancers: a systematic review. Nutrients. 2021;13:2842. 10.3390/nu13082842 34445002 PMC8400981

[85] Findlay M , Rankin NM, Shaw T, et al. Best evidence to best practice: implementing an innovative model of nutrition care for patients with head and neck cancer improves outcomes. Nutrients. 2020;12:1465. 10.3390/nu12051465 32438607 PMC7284331

[86] McIsaac DI , Gill M, Boland L, et al; Prehabilitation Knowledge Network. Prehabilitation in adult patients undergoing surgery: an umbrella review of systematic reviews. Br J Anaesth. 2022;128:244-257. 10.1016/j.bja.2021.11.014 34922735

[87] Mareschal J , Hemmer A, Douissard J, et al. Surgical prehabilitation in patients with gastrointestinal cancers: impact of unimodal and multimodal programs on postoperative outcomes and prospects for new therapeutic strategies—a systematic review. Cancers. 2023;15:1881. 10.3390/cancers15061881 36980767 PMC10047365

[88] Silver JK , Baima J. Cancer prehabilitation: an opportunity to decrease treatment-related morbidity, increase cancer treatment options, and improve physical and psychological health outcomes. Am J Phys Med Rehabil. 2013;92:715-727. 10.1097/PHM.0b013e31829b4afe 23756434

[89] Gillis C , Davies SJ, Carli F, et al. Current landscape of nutrition within prehabilitation oncology research: a scoping review. Front Nutr. 2021;8:644723. 10.3389/fnut.2021.644723 33898499 PMC8062858

[90] Orsso CE , Montes-Ibarra M, Findlay M, et al. Mapping ongoing nutrition intervention trials in muscle, sarcopenia, and cachexia: a scoping review of future research. J Cachexia Sarcopenia Muscle. 2022;13:1442-1459. 10.1002/jcsm.12954 35301816 PMC9178172

